# Three-Year Follow-Up of Posterior Chamber Toric Phakic Intraocular Lens Implantation for Moderate to High Myopic Astigmatism

**DOI:** 10.1371/journal.pone.0056453

**Published:** 2013-02-08

**Authors:** Kazutaka Kamiya, Kimiya Shimizu, Hidenaga Kobashi, Akihito Igarashi, Mari Komatsu

**Affiliations:** 1 Department of Ophthalmology, University of Kitasato School of Medicine, Kanagawa, Japan; 2 Sanno Hospital, Tokyo, Japan; Case Western Reserve University, United States of America

## Abstract

**Purpose:**

To assess the 3-year clinical outcomes of toric phakic intraocular lens (Visian ICL™; STAAR Surgical) implantation for moderate to high myopic astigmatism.

**Methods:**

This retrospective study evaluated fifty eyes of 28 patients who underwent toric ICL implantation for the correction of moderate to high myopic astigmatism and who regularly returned for postoperative examination. Before, and 1, 3, and 6 months after, and 1, 2, and 3 years after surgery, we assessed the safety, efficacy, predictability, stability, and adverse events of the surgery in eyes undergoing toric ICL implantation.

**Results:**

The logarithm of the minimal angle of resolution (LogMAR) uncorrected visual acuity and LogMAR best spectacle-corrected visual acuity were –0.10 (corresponding to Snellen equivalents 20/16) ± 0.16 and –0.20 (corresponding to 20/12.5) ± 0.07, 3 years postoperatively, respectively. The safety and efficacy indices were 1.16 ± 0.20 and 0.94 ± 0.28. At 3 year, 82% and 98% of the eyes were within 0.5 and 1.0 D, respectively, of the targeted correction. Manifest refraction changes of –0.15 ± 0.31 D occurred from 1 month to 3 year. No vision-threatening complications occurred during the observation period.

**Conclusions:**

On the basis of the clinical results of this study, toric ICL implantation was good in all measures of safety, efficacy, predictability, and stability for the correction of moderate to high myopic astigmatism throughout a 3-year observation period.

## Introduction

The Visian Implantable Collamer Lens (ICL™, STAAR Surgical, Nidau, Switzerland), a posterior chamber phakic intraocular lens (IOL) has been reported to be effective for the correction of moderate to high ametropia.[1]–[11] In addition, this surgical procedure is largely reversible and also the lens is exchangeable with another lens when unexpected refractive changes occur after surgery, unlike laser in situ keratomileusis (LASIK). Recently, toric ICL has also been demonstrated to be effective for the correction of high myopic astigmatism.[12]–[23] Since highly myopic eyes often show some astigmatism, implantation of a toric ICL may be a better surgical approach than that of a spherical ICL for correcting them. In consideration of the prevalence of this surgical procedure, it is essential to evaluate the long-term clinical outcomes of toric ICL implantation. We previously demonstrated that toric ICL implantation was good from the standpoints of safety, efficacy, predictability, and stability throughout the 1-year follow-up period, and that contrast sensitivity function was significantly improved after toric ICL implantation.[16] Matsumura et al reported in a preliminary study that toric ICL implantation was beneficial for the treatment of high myopic astigmatism, but neither a detailed time course analysis of visual and refractive outcomes nor astigmatic vector analysis were conducted in that study.[21] We extended the study in order to retrospectively investigate the long-term (3-year) clinical outcomes of toric ICL implantation in the correction of moderate to high myopia, in addition to astigmatic vector analysis.

## Materials and Methods

### Patient population

Fifty eyes (15 of men and 35 of women) of 28 patients, who underwent implantation of the posterior chamber toric phakic intraocular lens (Visian toric ICL™, STAAR Surgical) for the correction of moderate to high myopic astigmatism, and who regularly returned for postoperative examination and completed a 3-year follow-up, were included in this observational study. The sample size in this study offered 93% statistical power at the 5% level in order to detect a 0.10-difference in logarithm of the minimal angle of resolution (logMAR) of visual acuity, when the standard deviation (SD) of the mean difference was 0.20. The inclusion criteria for this surgical technique were as follows: unsatisfactory correction with spectacles or contact lenses, 20 ≤ age ≤ 50 years, stable refraction for at least 1 year, −3.0 to −20.0 diopters (D) of myopia with astigmatism of 0.75 D or more, anterior chamber depth ≥ 2.8 mm, endothelial cell density ≥ 1800 cells/mm^2^, no history of ocular surgery, progressive corneal degeneration, cataract, glaucoma or uveitis. The patient age at the time of surgery was 33.9 ± 7.7 years (mean age ± SD; range, 23 to 50 years). The preoperative manifest spherical equivalent was −9.47 ± 2.91 diopters (D) (range, −3.00 to −17.25 D). The preoperative manifest refractive cylinder was −2.23 ± 1.09 D (range, −0.75 to −6.50 D). Eyes with keratoconus were excluded from the study by using the keratoconus screening test of Placido disk videokeratography (TMS-2, Tomey, Nagoya, Japan). Before surgery and 1, 3, and 6 months, and 1, 2, and 3 years after surgery, we determined the following: logarithm of the minimal angle of resolution (logMAR) of uncorrected visual acuity (UCVA), logMAR of best spectacle-corrected visual acuity (BSCVA), manifest refraction (spherical equivalent), intraocular pressure (IOP), and endothelial cell density (except for 1 month postoperatively), in addition to the usual slit-lamp biomicroscopic and funduscopic examinations. The safety index was determined as the mean postoperative BSCVA / mean preoperative BSCVA, and the efficacy index as the mean postoperative UCVA / mean preoperative BSCVA. Before surgery, the horizontal white-to-white distance and anterior chamber depth were measured using a scanning-slit topograph (Orbscan IIz, Bausch & Lomb, Rochester, US), and the mean keratometric readings and the central corneal thickness were measured using an autorefractometer (ARK-700A, Nidek, Gamagori, Japan) and an ultrasound pachymeter (DGH-500, DGH Technologies, Exton, US), respectively. The IOP was assessed with a non-contact tonometer (KT-500, Kowa, Tokyo, Japan). The endothelial cell density was determined with a non-contact specular microscope (SP-8800, Konan, Nishinomiya, Japan). Slit-lamp evaluation of the alignment of the toric ICL was also performed after mydriasis, when UCVA was less than excellent, or manifest refraction was changed, or both. The study was approved by the Institutional Review Board at Kitasato University School of Medicine and followed the tenets of the Declaration of Helsinki. Informed written consent was obtained from all patients after explanation of the nature and possible consequences of the study.

### Toric implantable collamer lens power calculation

Toric ICL power calculation was performed by the manufacturer using the astigmatism decomposition method.[24] Toric ICLs were manufactured to minimize rotation, and required the surgeon to rotate the ICL no more than 22.5 degrees from the horizontal meridian. The size of the toric ICL was also chosen by the manufacturer on the basis of the horizontal corneal diameter and anterior chamber depth with scanning-slit topography (Orbscan IIz).

### Toric implantable collamer lens surgical procedure

The patients preoperatively underwent 2 peripheral iridotomies with a neodymium: YAG laser. On the day of surgery, the patients were administered dilating and cycloplegic agents. To control for potential cyclotorsion in a supine position, the zero horizontal axis was marked preoperatively using a slit-lamp. A Mendez ring was also used for measuring intraoperatively the required rotation from the horizontal axis. After topical anesthesia, a model V4 toric ICL was inserted through a 3-mm clear corneal incision with the use of an injector cartridge after placement of viscoelastic material (sodium hyaluronate 1%, Opegan™, Santen, Osaka, Japan) into the anterior chamber. After the ICL had then been placed in the posterior chamber and rotated by 22.5 degrees or less using the manipulator, the remaining viscoelastic material was completely washed out of the anterior chamber with balanced salt solution, and a miotic agent (acetylcholine chloride, Ovisot™, Daiichi-Sankyo, Tokyo, Japan) was instilled. All surgeries were uneventful and no definite intraoperative complication was observed. After surgery, steroidal (0.1% betamethasone, Rinderon™, Shionogi, Osaka, Japan) and antibiotic (0.5% levofloxacin, Cravit™, Santen, Osaka, Japan) medications were topically administered 4 times daily for 2 weeks at a dose that was steadily reduced.

### Power vector analysis

Spherocylindrical refraction results were converted to vectors expressed by 3 dioptric powers: M, J_0_, and J_45_, where M is equal to the spherical equivalent of the given refractive error and J_0_ and J_45_ are the 2 Jackson cross-cylinders equivalents to the conventional cylinder. Manifest refractions were recorded in conventional script notation (sphere, cylinder, and axis) and then converted to the power vector coordinates described by Thibos and Horner[25] and to overall blurring strength by the following formulas:

*M = S+C/2,*

*J_0_ = (-C/2)cos(2α), J_45_ = (-C/2)sin(2α),*

*B = (M^2^+J_0_^2^+J_45_^2^)^1/2^,*

where M is the spherical lens equal to the spherical equivalent of the given refractive error; S is the sphere; C is the cylinder; J_0_ is the Jackson cross-cylinder, axes at 180 degrees and 90 degrees; α is the axis; J_45_ is the Jackson cross-cylinder, axes at 45 degrees and 135 degrees; and B is the overall blurring strength of the spherocylindrical refractive error.

### Statistical analysis

All statistical analyses were performed using StatView version 5.0 (SAS, Cary, US). One-way analysis of variance (ANOVA) was used for the analysis of the time course of changes, with the Dunnett test for multiple comparisons. The Wilcoxon signed-rank test was used for statistical analysis to compare the pre- and post-surgical data. The results are expressed as mean ± standard deviation (SD), and a value of p<0.05 was considered statistically significant.

## Results

Preoperative patient demographics are summarized in Table 1. LogMAR UCVA and BSCVA were 1.49 ± 0.25 (range, 0.70 to 2.00) and −0.14 ± 0.06 (range, −0.30 to 0.00), respectively. Horizontal white-to-white distance was 11.7 ± 0.4 mm (range, 11.0 to 12.6 mm). Anterior chamber depth was 3.23 ± 0.25 mm (range, 2.85 to 3.74 mm). The mean keratometric reading was 43.8 ± 1.5 D (range, 41.1 to 47.1 D). Central corneal thickness was 530.4 ± 31.4 µm (range, 471 to 600 µm). The IOP was 13.4 ± 2.6 mmHg (range, 8.8 to 21.0 mmHg). The endothelial cell density was 2753 ± 284 cells/mm^2^ (range, 1828 to 3333 cells/mm^2^).

**Table 1:** Preoperative patient demographics of the study population.

Characteristic	Mean ± Standard Deviation
Age (years)	33.9 ± 7.7 years (range, 23 to 50 years)
Gender (% female)	64.3%
Manifest spherical equivalent (D)	−9.47 ± 2.91 (range, −3.00 to −17.25 D)
Manifest cylinder (D)	−2.23 ± 1.09 D (range, −0.75 to −6.50 D)
Log MAR UCVA	1.49 ± 0.25 (range, 0.70 to 2.00)
Log MAR BSCVA	−0.14 ± 0.06 (range, −0.30 to 0.00)
White-to-white distance (mm)	11.7 ± 0.4 mm (range, 11.0 to 12.6 mm)
Anterior chamber depth (mm)	3.23 ± 0.25 mm (range, 2.85 to 3.74 mm)
Mean keratometric readings (D)	43.8 ± 1.5 D (range, 41.1 to 47.1 D)
Central cornea thickness ( µm)	530.4 ± 31.4 µm (range, 471 to 600 µm)
Log MAR = logarithm of the minimal angle of resolution, UCVA = uncorrected visual acuity	
BSCVA = best spectacle-corrected visual acuity	

### Safety outcomes

**Figure 1:**
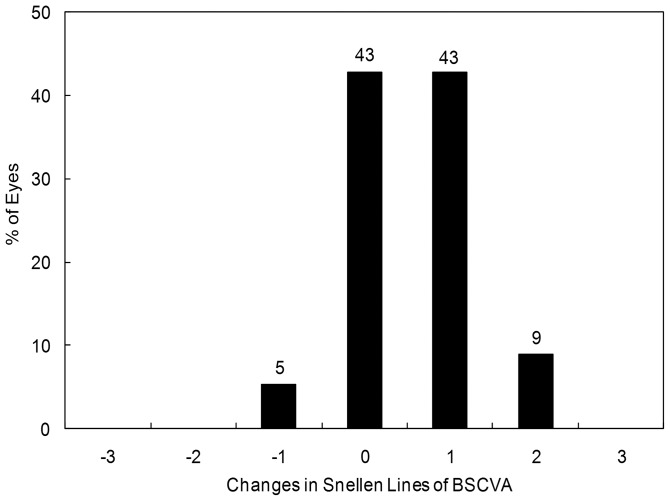
Changes in best spectacle-corrected visual acuity (BSCVA) after toric implantable collamer lens implantation. BSCVA =  best spectacle-corrected visual acuity

LogMAR BSCVA was −0.22 ± 0.07, −0.22 ± 0.07, −0.22 ± 0.07, −0.23 ± 0.08, −0.21 ± 0.08, and −0.20 (corresponding to Snellen equivalents 20/12.5) ± 0.07, 1, 3, and 6 months, and 1, 2, and 3 years after surgery, respectively (ANOVA, p = 0.54). The safety index over similar periods was 1.19 ± 0.20, 1.21 ± 0.21, 1.21 ± 0.20, 1.23 ± 0.22, 1.17 ± 0.21, and 1.16 ± 0.20. Twenty-four eyes (48%) showed no change in BSCVA, 22 eyes (44%) gained 1 line and 2 eyes (4%) gained 2 lines, 2 eyes (4%) lost 1 line, and no eyes lost 2 or more lines 3 years after toric ICL implantation (Figure 1). Ten degrees or more of axis rotation, deteriorating UCVA or changing refraction, occurred in 6 eyes (12%) postoperatively, and all required ICL repositioning.

### Effectiveness outcomes

**Figure 2:**
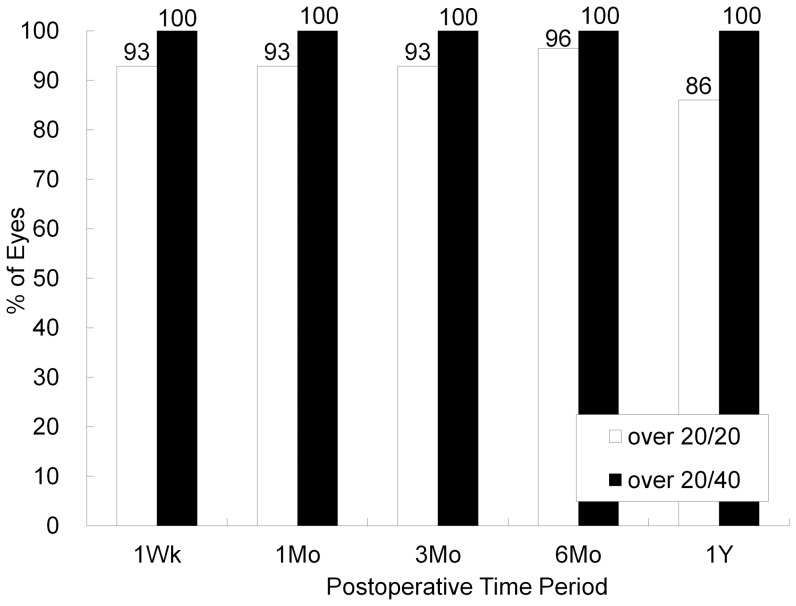
Changes in uncorrected visual acuity (UCVA) after toric implantable collamer lens implantation. UCVA = uncorrected visual acuity

LogMAR UCVA was −0.13 ± 0.12, −0.14 ± 0.12, −0.15 ± 0.11, −0.15 ± 0.13, −0.12 ± 0.17, and −0.10 (corresponding to Snellen equivalents 20/16) ± 0.16, 1, 3, and 6 months, and 1, 2, and 3 years after surgery, respectively (ANOVA, p = 0.42). The efficacy index over similar periods was 1.00 ± 0.25, 1.02 ± 0.25, 1.03 ± 0.24, 1.03 ± 0.25, 0.98 ± 0.30, and 0.94 ± 0.28. At 1, 3, and 6 months, and 1, 2, and 3 years after surgery, 100%, 100%, 100%, 100%, 96%, and 98% of eyes, and 94%, 94%, 92%, 90%, 84%, and 86% of eyes had a UCVA of 20/40, and of 20/20 or better, respectively (Figure 2).

### Predictability

**Figure 3:**
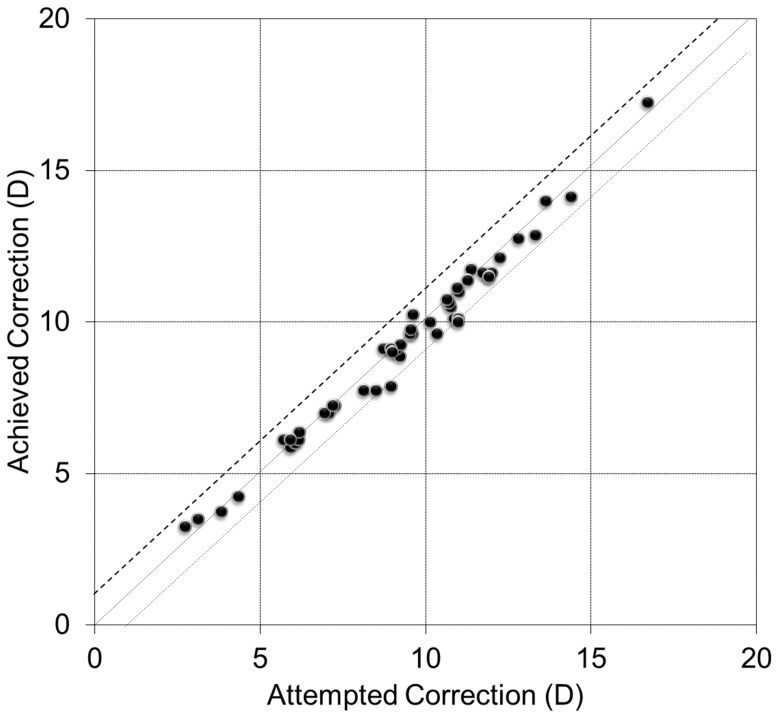
Scatter plot of attempted vs achieved correction (spherical equivalent) after toric implantable collamer lens implantation. D = diopter

A scatter plot of the attempted versus the achieved correction (spherical equivalent) is shown in Figure 3. At 3 years after surgery, 82 and 98% of eyes were within ± 0.5 and ± 1.0 D of the attempted spherical equivalent correction, respectively. At 3 years after surgery, 92 and 94% of eyes were within ± 0.5 and ± 1.0 D of the attempted astigmatic correction, respectively.

### Stability

#### Spherical equivalent

**Figure 4:**
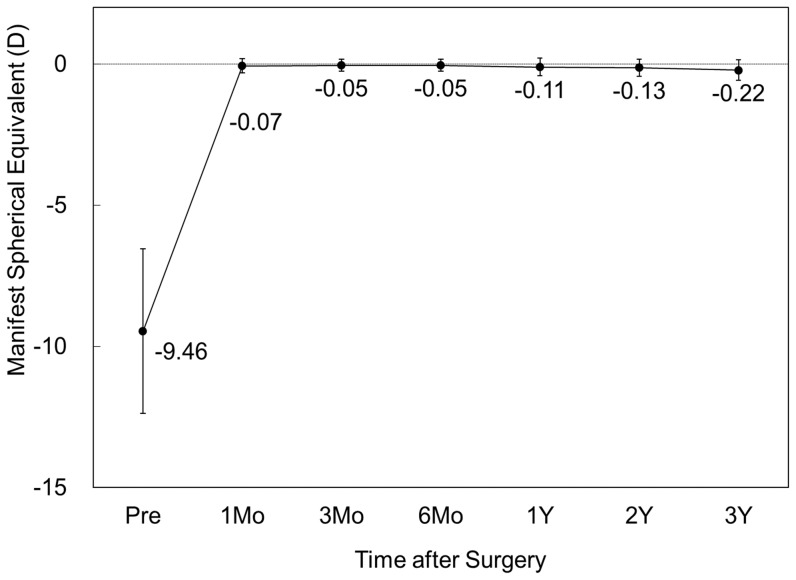
Time course of manifest spherical equivalent after toric implantable collamer lens implantation. D = diopter, M = month, Y = year

The change in the manifest spherical equivalent is shown in Figure 4. At 1, 3, and 6 months, and 1, 2, and 3 years after surgery, the mean manifest spherical equivalent was −0.07 ± 0.25, −0.05 ± 0.22, −0.05 ± 0.21, −0.11 ± 0.32, −0.13 ± 0.30, and −0.22 ± 0.37 D, respectively (ANOVA, p = 0.02). Multiple comparisons demonstrated a significant difference between measurements made at 1 month after and at 3 years after (p = 0.04, Dunnett test). Changes in manifest refraction from 1 month to 3 months, from 3 months to 6 months, from 6 months to 1 year, from 1 year to 2 years, and from 2 years to 3 years were 0.02 ± 0.16, 0.00 ± 0.17, −0.06 ± 0.28, −0.03 ± 0.21, and −0.09 ± 0.25 D, respectively.

#### Astigmatism

**Figure 5:**
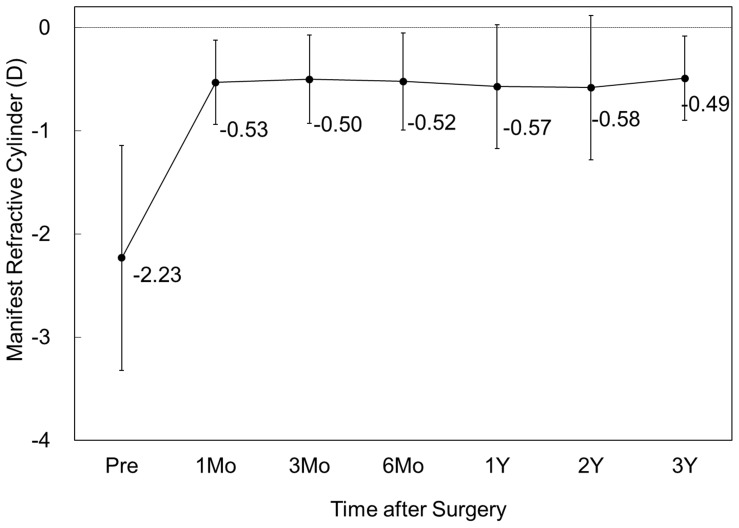
Time course of manifest refractive cylinder after toric implantable collamer lens implantation. D = diopter, M = month, Y = year

The change in the manifest refractive cylinder is shown in Figure 5. At 1, 3, and 6 months, and 1, 2, and 3 years after surgery, the mean manifest refractive cylinder was −0.53 ± 0.41, −0.50 ± 0.43, −0.52 ± 0.47, −0.57 ± 0.60, −0.58 ± 0.70, and −0.49 ± 0.41 D, respectively (ANOVA, p = 0.92). Changes in manifest refractive cylinder 1 month to 3 months, from 3 months to 6 months, from 6 months to 1 year, from 1 year to 2 years, and from 2 years to 3 years were 0.03 ± 0.24, −0.02 ± 0.24, −0.05 ± 0.38, −0.01 ± 0.30, and −0.10 ± 0.58 D, respectively.

### Intraocular pressure

**Figure 6:**
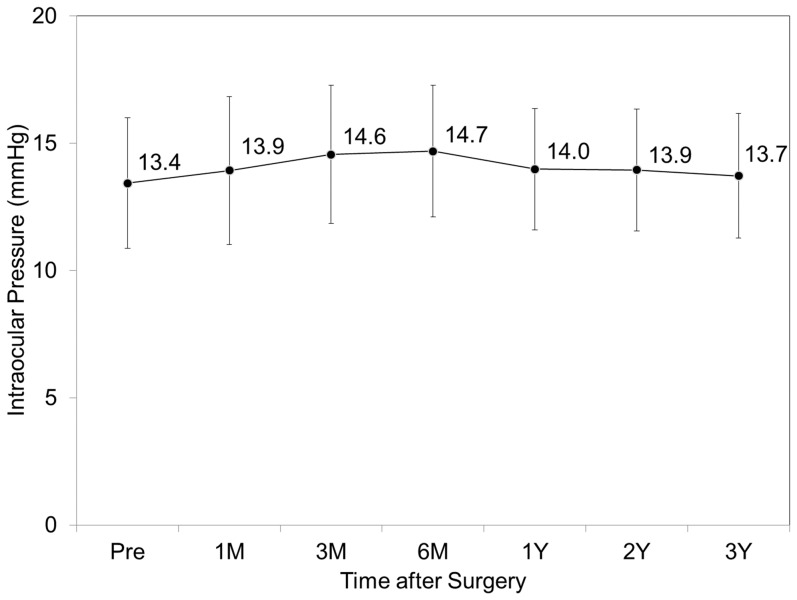
Time course of intraocular pressure after toric implantable collamer lens implantation. M = month, Y = year

The IOP was 13.9 ± 2.9, 14.6 ± 2.7, 14.7 ± 2.6, 14.0 ± 2.4, 13.9 ± 2.4, and 13.7 ± 2.4 mmHg, 1, 3, and 6 months, and 1, 2, and 3 years after surgery, respectively (ANOVA, p = 0.18) (Figure 6). No significant increase in IOP (> 23 mmHg) occurred in any case during the observation period.

### Endothelial cell density

**Figure 7:**
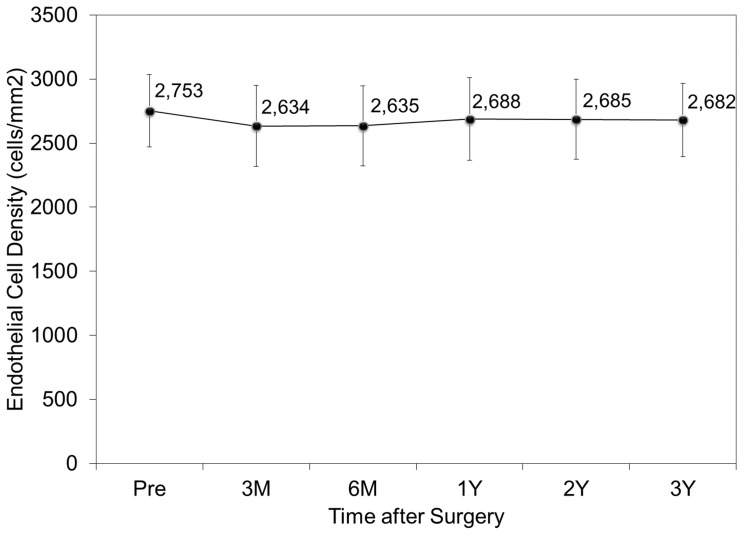
Time course of endothelial cell density after toric implantable collamer lens implantation. M = month, Y = year

The endothelial cell density fell from 2753 ± 284 cells/mm^2^ preoperatively to 2634 ± 318, 2635 ± 312, 2688 ± 323, 2685 ± 313, and 2682 ± 286 cells/mm^2^, 3 and 6 months, and 1, 2, and 3 years postoperatively (ANOVA, p = 0.41) (Figure 7). The mean percentage of endothelial cell loss was 2.3% 3 years postoperatively.

### Power vector analysis

**Figure 8:**
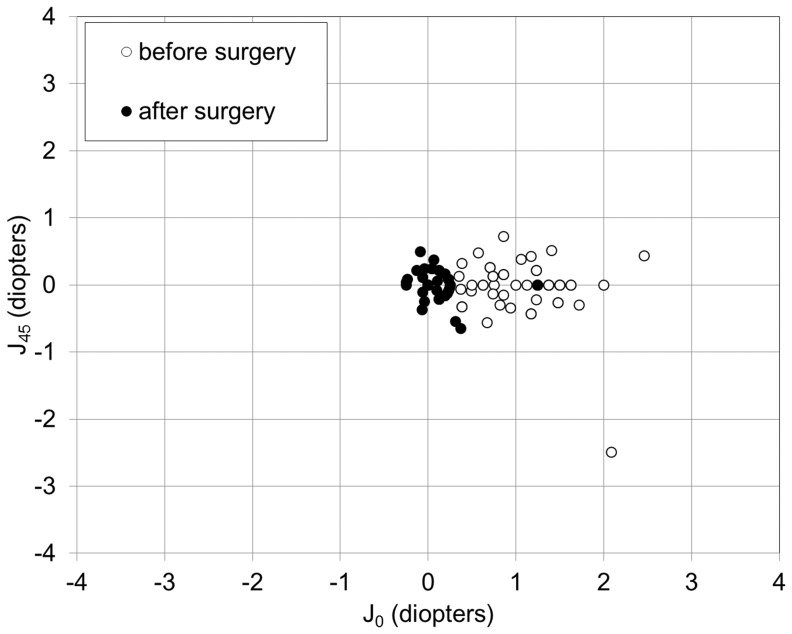
Power vector analysis. Preoperative and postoperative (at 3 years) manifest refractive astigmatism, plotted as an astigmatic vector for each eye, referenced to the spectacle plane.

The changes in the astigmatic power vector between preoperative and postoperative (after 3 years) values for all cases are presented in Figure 8. The dispersed cluster of points before surgery tended to collapse around the origin after surgery, indicating a reduction in vector astigmatic change. The distribution of the manifest refractive errors after vector conversion before and 3 years after surgery is shown in Table 2. For J_0_, 98% of eyes were within ± 0.5 D, and 98% were within ± 1.0 D. For J_45_, 96% of eyes were within ± 0.5 D, and 100% were within ± 1.0 D, 3 years after toric ICL implantation.

**Table 2:** Refraction after vectorial conversion before and 3 years after toric implantable collamer lens implantation.

Refraction	Before surgery	After surgery
M	−9.47 ± 2.91 D (range, −3.00 to −17.25 D)	−0.22 ± 0.37 D (range, −1.25 to 0.50 D)
J_0_	1.05 ± 0.49 D (range, 0.25 to 2.46 D)	0.10 ± 0.22 D (range, −0.25 to 1.25 D)
J_45_	−0.04 ± 0.44 D (range, −2.49 to 0.72 D)	0.01 ± 0.21 D (range, −0.65 to 0.49 D)
B	9.57 ± 2.82 D (range, 3.91 to 17.32 D)	0.42 ± 0.32 D (range, 0.00 to 1.27 D)

D  =  diopter, M  =  spherical equivalent refraction, J_0_  =  Jackson cross-cylinder, axes at 0 and 90 degrees, J_45_ =  Jackson cross-cylinder, axes at 45 and 135 degrees, B  =  blur strength

### Secondary surgeries / adverse events

There were no intraoperative complications, and all implantations were uneventful. In 50 eyes, four eyes (8%) required ICL repositioning (in 3 eyes 1 day postoperatively, and in 1 eye 2 years postoperatively) because of axis rotation by 10 degrees or more. Another 2 eyes (4%) also required this repositioning because of a traumatic event. In 50 eyes, four eyes (8%) developed asymptomatic anterior subcapsular cataract (in 2 eyes 1 day postoperatively, in 1 eye 3 months postoperatively, and in 1 eye 1 year postoperatively), in which 3 eyes (6%) showed no change in BSCVA, and 1 eye (2%) lost 1 line. No eyes developed symptomatic anterior subcapsular cataract reaching as far as the axial area of the eye. Simultaneous ICL extraction and cataract surgery was not necessary because all these eyes had 20/16 or more of BSCVA. Otherwise, neither pigment dispersion glaucoma, nor pupillary block, nor any other vision-threatening complication was seen at any time during the 3-year follow-up period.

## Discussion

Judged on the basis of the clinical results of the present study, toric ICL implantation performed well for the correction of moderate to high myopic astigmatism throughout the 3-year follow-up period. Since the number of the complications of ICL implantation such as cataract formation, endothelial cell loss, pigment dispersion syndrome, pigmentary glaucoma, and pupillary block are expected to increase with time,[1]–[11] it is of clinical importance to elucidate the long-term outcomes for the prevalence of this surgical approach. However, many studies have focused on the short-term clinical outcomes after up to 6 months to 1 year.[12]–[22] To the best of our knowledge, the present work is one of the longest-term published studies to assess the time course of visual and refractive outcomes of toric ICL implantation and astigmatic vector change for moderate to high myopic astigmatism. The United States Food and Drug Administration (U.S. FDA) Toric ICL 1-year clinical study demonstrated that toric ICL implantation was an effective and predictable method for treating moderate to high myopic astigmatism.[12] We and Bhikoo et al previously demonstrated that toric ICL implantation was successful from the standpoints of safety, efficacy, predictability, and stability over a 1-year observation period.[15], [16] Sari et al retrospectively assessed the 3-year clinical outcomes of toric ICL implantation, and reported that 17.6% of the implanted eyes showed no change in BSCVA, while 76.5% gained up to 2 lines and 5.9% lost 1 line, which was essentially equivalent to our safety outcomes.[23] They also reported that 52.9% of the eyes had a spherical equivalent within ±0.5D, and that 82.4% of the eyes within ±1.0 D, of emmetropia with a slight tendency toward undercorrection 3 years after toric ICL implantation were slightly worse than those in our predictability outcomes. Schallhorn et al reported that the toric ICL performed better than photorefractive keratectomy in terms of safety, efficacy, predictability and stability.[13] We previously reported that toric ICL implantation was better than wavefront-guided LASIK, which appeared to be one of best surgical techniques in corneal refractive surgery, in almost all measures of safety, efficacy, predictability, and stability.[14] Choi et al stated that toric ICL implantation provided a more stable visual outcome than bioptics (ICL and excimer laser ablation) and led to the elimination of laser treatments and their inherent risks.[20] Alfonso et al recently reported that both toric ICL implantation and bioptics (ICL implantation plus excimer corneal surgery) showed good clinical results in patients with myopic astigmatism, reducing preoperative spherical and astigmatic errors with high predictability and safety, although the bioptic procedures showed slightly better outcomes in some clinical measures.[22] Hence, we believe that toric ICL implantation has advantages over keratorefractive surgical techniques, and that these advantages are more marked with higher myopic and astigmatic eyes requiring greater laser ablation. It is likely that this surgical approach through a 3.0-mm corneal incision, regardless of the amount of myopic correction, is less subject to the wound healing responses of the cornea, unlike keratorefractive surgical procedures.

Toric lens-specific complications such as axis rotation after surgery need to be determined over a prolonged observation period, because this new lens can induce rotation of the ICL, which may result in the deterioration of visual performance and consequently, patient satisfaction. When visual performance was excellent, dilation was not performed in order to measure the exact ICL position in the current study. We therefore have no data on toric lens stability in the population as a whole, only in those patients with suboptimal optics. Forty-four of 50 eyes (88%) showed excellent UCVA throughout the 3-year observation period, suggesting that no significant improper alignment or axis rotation occurred, at least in such eyes. However, 4 eyes (8%) required ICL repositioning because of spontaneous axis rotation, and another 2 eyes (4%) because ocular trauma occurring in the early postoperative period. Sari et al showed that toric ICL repositioning was needed in 3 eyes (8.8%) 1 day after surgery.[23] We currently use ICL sizes ranging from 11.5 to 13.0 mm in steps of 0.5 mm, and researchers have usually selected one of these sizes on the basis only of horizontal corneal diameter and anterior chamber depth. Extreme underestimation or overestimation of ICL size was not likely to occur in these eyes, because no excessively low or high vault was observed between the ICL and the crystalline lens. Additionally, no significant axis rotation recurred after the ICL repositioning. Therefore, we assume that the ICL haptics were fixated, not in the sulcus, but in the ciliary body, resulting in axis rotation of the ICL due to physiological pupil movements or a traumatic event. Ultrasound biomicroscopy may be useful for detailed analysis in order to confirm whether or not the position of the ICL affects axis rotation after this surgery. It should be emphasized that a toric ICL can be easily and safely repositioned without any intraoperative complications even if improper alignment or rotation of the axis of the ICL occurs in the late postoperative period.

With regard to astigmatic correction, Alfonso et al reported, in a study of 15 high astigmatic eyes, that 93.3% of eyes were within ±1.0 D of J0 and that all eyes were within ±1.0 D of J45 for their astigmatic components.[17] They also reported, in a study of 55 toric ICL-implanted eyes with myopic astigmatism, that for J0, 94.5% of eyes, and for J45, 98.2% of eyes, were within ±0.5 D, and that all eyes were within ±1.0 D.[18] It was also demonstrated, in a study of 30 toric ICL-implanted eyes with keratoconus, that 83.3% and 86.7% of eyes were within ±0.5 D for the astigmatic components J0 and J45, respectively.[19] Toric ICL power has hitherto been calculated on the assumption that surgically induced astigmatism is negligible. Our results indicate that manifest astigmatism was significantly reduced, but that approximately 0.5 D of astigmatic error remained after surgery. We previously showed that spherical ICL implantation induced corneal astigmatism through a with-the-rule astigmatic shift of approximately 0.5 D, which was small but not negligible for refractive surgery candidates.[26] Since the 3.0-mm corneal incision used in toric ICL implantation is identical to that for spherical ICL implantation, we assume that the remaining manifest astigmatism can be largely attributed to the astigmatism induced by this surgical technique through the 3.0-mm incision.

There are ongoing concerns both about the development of lens opacity because of the close proximity of the ICL to the crystalline lens and about endothelial cell loss after the implantation of any ICL. With regard to the long-term onset of cataract formation after ICL implantation, Fernandes et al reported, after a literature search of the PubMed database, that a total of 136 (5.2%) of 2592 eyes developed cataract after ICL implantation.[27] Lackner et al reported that 11 eyes (14.5%) developed lens opacification 3 years postoperatively.[7] The U.S. FDA Trial demonstrated that the incidence of anterior subcapsular cataracts with ICL V4 was 2.7% 3 years postoperatively.[8] Subsequently, Sanders et al also reported in the U.S. FDA trial that 6% to 7% of eyes develop anterior subcapsular opacities 7 years after ICL implantation, but that only 1% to 2% progress to visually significant cataracts during the same period.[28] We previously reported that 6 eyes (11%) developed asymptomatic anterior subcapsular cataracts, and that 1 eye (2%) developed a clinically significant symptomatic anterior subcapsular cataract 4 years after spherical ICL implantation.[10] Alfonso et al stated that 3 eyes (1.6%) developed late anterior subcapsular cataracts, one of which was visually significant, leading to ICL removal and phacoemulsification.[11] In this study, during the 3-year follow-up, we noticed 4 eyes (8%) in which asymptomatic cataracts developed, but none in which symptomatic cataracts appeared, but ICL extraction was not required because all of these eyes had 20/16 or more of BSCVA. The incidence of cataract formation in the present study was comparable with that in previous studies,[7], [10], [28] except for one study,[11] after spherical ICL implantation. Sari et al reported that 2 eyes (5.8%) developed anterior subcapsular lens opacities not affecting the final BSCVA 3 years after toric ICL implantation.[23] It is suggested that the additional maneuver performed while the haptics are behind the iris does not directly cause cataract formation.

With regard to long-term endothelial cell loss after ICL implantation, Fernandes et al stated that the mean endothelial cell loss varied from 9.9% at 2 years to 3.7% 4 years postoperatively, and that this loss was more pronounced within the first 1 to 2 years, with stability or lower progression after that time.[27] Jiménez-Alfaro reported that the percentage of endothelial cell loss was 6.57% 2 years after surgery.[3] Lackner et al stated that the endothelial cell density was slightly decreased in eyes with clear lenses, but that the decrease was more pronounced in those in which opacification occurred.[7] The U.S. FDA Trial demonstrated that the endothelial cell loss was 8.4 to 9.7% 3 years postoperatively.[8] Pineda-Fernandez reported 6.09% of endothelial cell loss 3 years after surgery.[9] Also, we found that the mean percentage of endothelial cell loss was 3.7% 4 years postoperatively,[10] and Alfonso et al stated that the total endothelial cell loss was 7.7% 5 years after surgery.[11] Sari et al reported that the mean percentage of endothelial cell loss was 8.7% 3 years after toric ICL implantation.[23] In the current study, the mean percentage of endothelial cell loss was 2.3% 3 years postoperatively, which was considerably lower than the findings in previous studies. [3], [7]–[11], [23] Considering that the surgical procedures of toric ICL implantation are almost identical to those of spherical ICL implantation, we assume that the differences of follow-up time, sample size, surgeon's skill, or other patient background factors such as age, as well as the reproducibility obtained with a noncontact specular microscope may be involved in this discrepancy.

With regard to the IOP rise after ICL implantation, we found no significant IOP rise throughout the 3-year follow-up time. However, an early rise in IOP was reported to be relatively frequent and usually moderate (≤30 mmHg).[27] Acute pupillary block and subsequent narrowing of the iridocorneal angle are considered primary causes of sustained elevated IOP, frequently associated with inadequate preoperative iridotomies and/or excessive ICL vault. A careful IOP monitoring is necessary to establish the long-term safety of this surgical procedure.

In conclusion, our clinical results may support the view that the toric ICL performs well in correcting moderate to high myopic astigmatism throughout the 3-year observation period. In addition, neither significant endothelial cell loss, significant IOP rise, nor vision-threatening complications occurred throughout the 3-year follow-up time. These findings suggest that toric ICL implantation may become a viable alternative to corneal refractive procedures for the treatment of such eyes. Further studies with a far greater number of subjects are required in order to confirm these findings.
